# Unified Monogamy Relations of Multipartite Entanglement

**DOI:** 10.1038/s41598-019-52817-y

**Published:** 2019-11-11

**Authors:** Awais Khan, Junaid ur Rehman, Kehao Wang, Hyundong Shin

**Affiliations:** 1 Department of Electronic Engineering, Kyung Hee University, Yongin-si, 17104 Korea; 2 Hubei Key Laboratory of Broadband Wireless Communication and Sensor Networks, Wuhan University of Technology, Wuhan, 430070 China

**Keywords:** Quantum information, Qubits

## Abstract

Unified-(*q*, *s*) entanglement $({{\mathscr{U}}}_{q,s})$ is a generalized bipartite entanglement measure, which encompasses Tsallis-*q* entanglement, Rényi-*q* entanglement, and entanglement of formation as its special cases. We first provide the extended (*q*; *s*) region of the generalized analytic formula of  ${{\mathscr{U}}}_{q,s}$. Then, the monogamy relation based on the squared  ${{\mathscr{U}}}_{q,s}$ for arbitrary multiqubit mixed states is proved. The monogamy relation proved in this paper enables us to construct an entanglement indicator that can be utilized to identify all genuine multiqubit entangled states even the cases where three tangle of concurrence loses its efficiency. It is shown that this monogamy relation also holds true for the generalized W-class state. The αth power ${{\mathscr{U}}}_{q,s}$ based general monogamy and polygamy inequalities are established for tripartite qubit states.

## Introduction

Entanglement is a vital asset in quantum information sciences that can enhance quantum technologies such as communication, cryptography and computing beyond classical limitations^1^. Such quantum technologies mostly rely on the distribution of entanglement in multipartite settings. Quantification and characterization of entanglement distribution for multipartite systems is well explained through monogamy relation. Briefly, the monogamy explains that if two parties are maximally entangled, then the rest of the parties cannot share any entanglement with them. This monogamy property, for example, plays a role in security analysis of quantum key distribution^2^ and it can also be used to distinguish quantum channels^3^.

The concept of monogamy of entanglement was first introduced by Coffman, Kundu and Wootters^4^–known as *CKW inequality*. They established the monogamy property for tripartite (*A*, *B*, and *C*) system via an entanglement measure called the concurrence^5^. Furthermore, the monogamy inequality asserts that the summation of individual entanglement content of subsystem *A* with subsystem *B* and with subsystem *C* is less than or equal to the entanglement of subsystem *A* with combined subsystem *BC*. This monogamy relation was then generalized to *N*-qubit systems^6^. Later on, monogamy relations for various entanglement measures have been proved, e.g., concurrence^4,7–9^, entanglement of formation^6,10,11^, negativity^9,12–15^, Tsallis-*q* entanglement^16–18^, and Rényi-*q* entanglement^19,20^. The dual of monogamy (polygamy) relation via the concurrence of assistance was proposed to quantify the limitation of distributing bipartite entanglement in multipartite systems^21,22^. Polygamy relations were established using various entanglement measures, e.g., convex-roof extended negativity^13^, and Tsallis-*q* entanglement^9,16^.

This paper proposes the idea to understand the entanglement distribution in multipartite system via the unified-(*q*, *s*) entanglement  $({{\mathscr{U}}}_{q,s})$.  ${{\mathscr{U}}}_{q,s}$ encompasses several measures of entanglement such as concurrence, Tsallis-*q* entanglement (T_*q*_ -E), Rényi-*q* entanglement (R_*q*_-E), and entanglement of formation (EOF), as its special cases. However, it does not satisfy the usual monogamy relations and violates monogamy for W-class state^23^. The monogamy relation of EOF has been not reported yet in a unified fashion. Three tangle based on the squared concurrence also has some flaws for entanglement detection^24^. This highly motivates us to introduce a general concept of monogamy relations in multiqubit systems, which can overcome these flaws. We propose new monogamy relations for ${{\mathscr{U}}}_{q,s}$. To this end, we first give the analytic formula of  ${{\mathscr{U}}}_{q,s}$ for the region $q\ge (\sqrt{9{s}^{2}-24s+28}-(2+3s))/(2(2-3s))$, 0 ≤ *s* ≤ 1, and $qs\le (5+\sqrt{13})/2$. Then, we establish the monogamy relation of multiqubit entangled system based on the squared ${{\mathscr{U}}}_{q,s}$ (SU-(*q*,*s*)-E), which encompasses the monogamy relations of EOF, T_*q*_-E, and R_*q*_-E, as special cases. Therefore, the results in this paper provide a unifying framework for monogamy relations in multiqubit systems, covering several previous monogamy results^6,16–20,23^.

## Results

First, we revise the definition of ${{\mathscr{U}}}_{q,s}$ and present the formula with its extended ranges. Then we investigate the monogamy relations for the squared and *α* ≥ 2 power of  ${{\mathscr{U}}}_{q,s}$. Polygamy relation of ${{\mathscr{U}}}_{q,s}$ for *α* ≤ 0 is also obtained. We further construct the multipartite entanglement indicator and present some numerical examples.

### Unified-(*q*,*s*) entanglement

For any bipartite pure state ${|\psi \rangle }_{AB}$, ${{\mathscr{U}}}_{q,s}$ is defined as^23^

1 $${{\mathscr{U}}}_{q,s}({|\psi \rangle }_{AB})={(s\mathrm{(1}-q))}^{-1}[{({\rm{tr}}{\rho }_{A}^{q})}^{s}-1],$$

for (*q*,*s*) ≥ 0|_*q*≠1,*s*≠0_, where the state of the subsystem *A* is obtained by tracing out the subsystem *B*, i.e., ${\rho }_{A}={{\rm{tr}}}_{B}[{|\psi \rangle }_{AB}\langle \psi |]$.

For any bipartite mixed state *ρ*_*AB*_, ${{\mathscr{U}}}_{q,s}$ and ${{\mathscr{U}}}_{q,s}$ of assistance $({\hat{{\mathscr{U}}}}_{q,s})$ are defined as

2 $${{\mathscr{U}}}_{q,s}({\rho }_{AB})=\,{\rm{\min }}\sum _{i}\,{p}_{i}{{\mathscr{U}}}_{q,s}({|{\psi }_{i}\rangle }_{AB}),$$

3 $${\hat{{\mathscr{U}}}}_{q,s}({\rho }_{AB})=\,{\rm{\max }}\sum _{i}\,{p}_{i}{{\mathscr{U}}}_{q,s}({|{\psi }_{i}\rangle }_{AB}),$$

where the minimization and maximization are obtained over all pure state decompositions ${\sum }_{i}\,{p}_{i}{|{\psi }_{i}\rangle }_{AB}\langle {\psi }_{i}|$ of *ρ*_*AB*_.

The ${{\mathscr{U}}}_{q,s}$ encompasses various entanglement measures depending on the parameters *q* and *s*. For example, it converges to R_*q*_-E, T_*q*_-E, and EOF when *s* → 0, *s* → 1, and *q* → 1, respectively.

### Refining the analytical formula for ${{\mathscr{U}}}_{q,s}$

For any two-qubit mixed state *ρ*_*AB*_, concurrence ${\mathscr{C}}$ is given as^5^

4 $${\mathscr{C}}({\rho }_{AB})=\,{\rm{\max }}\{\mathrm{0,}{\mu }_{1}-{\mu }_{2}-{\mu }_{3}-{\mu }_{4}\},$$

where *μ*_*i*_ are the decreasing eigenvalues of $\sqrt{{\rho }_{AB}({\sigma }_{{\rm{y}}}\otimes {\sigma }_{{\rm{y}}}){\rho }_{AB}^{\ast }({\sigma }_{{\rm{y}}}\otimes {\sigma }_{{\rm{y}}})}$, and *σ*_y_ denotes the Pauli-y operator.

The analytic relationship between ${{\mathscr{U}}}_{q,s}$ and concurrence of a bipartite state *ρ*_*AB*_ for 1 ≥ *s* ≥ 0 and 3/*s* ≥ *q* ≥ 1 has been unveiled as follows^23^:

5 $${{\mathscr{U}}}_{q,s}({\rho }_{AB})={f}_{q,s}({\mathscr{C}}({\rho }_{AB})),$$

where

6 $${f}_{q,s}(x)=\frac{{({\eta }_{+}^{q}+{\eta }_{-}^{q})}^{s}-{2}^{qs}}{s(1-q){2}^{qs}}$$

with $${\eta }_{\pm }=(1\pm \sqrt{1-{x}^{2}})$$.

The analytic formula (5) holds until the *f*_*q*,*s*_(*x*) in (6) is monotonically increasing and convex for any *q* and *s* value^23^. The monotonicity and convexity follow from the fact that ∂*f*_*q*,*s*_(*x*)/∂*x* ≥ 0 for all *q* ≥ 0 and ∂^2^*f*_*q*,*s*_(*x*)/∂*x*^2^ ≥ 0 for 1 ≥ *s* ≥ 0 and 3/*s* ≥ *q* ≥ 1^23^.

In the Methods section, we prove that $${f}_{q,s}({\mathscr{C}})$$ is a convex function of $${\mathscr{C}}$$ for the region $$q\ge (\sqrt{9{s}^{2}-24s+28}-$$$$(2+3s))$$/(2(2 − 3*s*)), 0 ≤ *s* ≤ 1, and $$qs\le (5+\sqrt{13})/2$$. Therefore, we have an extended (*q*, *s*)-region with $$q\ge (\sqrt{9{s}^{2}-24s+28}-(2+3s))$$/(2(2 − 3*s*)), 0 ≤ *s* ≤ 1, and $$qs\le (5+\sqrt{13})/2$$, where the second-order derivative of *f*_*q*,*s*_(*x*) is nonnegative. Consequently, the analytic formula of unified-(*q*, *s*) entanglement (5) now holds for

$${\mathscr{R}}=\{(q,s)|(\sqrt{9{s}^{2}-24s+28}-(2+3s))/(2(2-3s))\le q\le (5+\sqrt{13})/2s,0\le s\le 1\}.$$

### Monogamy relation for SU-(*q*,*s*)-E in multiqubit systems

The main result of the paper is the general monogamy inequality of SU-(*q*,*s*) -E ${{\mathscr{U}}}_{q,s}^{2}$ for an arbitrary multipartite qubit mixed state (see Theorem 1), i.e.,

7 $${{\mathscr{U}}}_{q,s}^{2}({\rho }_{A{B}_{1}{B}_{2}\cdots {B}_{N-1}})-{{\mathscr{U}}}_{q,s}^{2}({\rho }_{A{B}_{1}})-{{\mathscr{U}}}_{q,s}^{2}({\rho }_{A{B}_{2}})-\cdots -{{\mathscr{U}}}_{q,s}^{2}({\rho }_{A{B}_{N-1}})\ge \mathrm{0,}$$

where ${{\mathscr{U}}}_{q,s}^{2}({\rho }_{A{B}_{1}{B}_{2}\cdots {B}_{N-1}})$ quantifies entanglement in the partition *A*|*B*_1_*B*_2_ … *B*_*N*−1_, and ${{\mathscr{U}}}_{q,s}^{2}({\rho }_{A{B}_{i}})$ quantifies the bipartite entanglement between *A* and *B*_*i*_. Before approaching towards our main relations, we propose two propositions, whose proofs are given in Methods section. These propositions are used for establishing the monogamy relation of ${{\mathscr{U}}}_{q,s}$.

We define

8 $${{\mathscr{U}}}_{q,s}({|\psi \rangle }_{AB})={g}_{q,s}({{\mathscr{C}}}^{2}({|\psi \rangle }_{AB}))=\frac{{({(1+\sqrt{1-{{\mathscr{C}}}^{2}})}^{q}+{(1-\sqrt{1-{{\mathscr{C}}}^{2}})}^{q})}^{s}-{2}^{qs}}{(1-q)s{2}^{qs}}.$$

**Proposition 1**. *SU-*(*q*,*s*)-*E* ${g}_{q,s}^{2}({{\mathscr{C}}}^{2})$ with $(q,s)\in  {\mathcal R}$ varies monotonically as a function of squared concurrence ${{\mathscr{C}}}^{2}$.

**Proposition 2**. *SU-*(*q*,*s*)-*E* ${g}_{q,s}^{2}({{\mathscr{C}}}^{2})$ with $(q,s)\in  {\mathcal R}$ is convex as a function of squared concurrence ${{\mathscr{C}}}^{2}$.

In the succeeding theorem, we will establish the monogamy inequity of ${{\mathscr{U}}}_{q,s}^{2}$ for *N*-qubit mixed state *ρ*_*AB*__1__*B*__2__…__*B*__*N*__−1_.

**Theorem 1**. *SU-*(*q*,*s*)-*E holds the following monogamy inequality for an arbitrary multi-qubit mixed state* *ρ*_*AB*__1__*B*__2__…__*B*__*N*__−1_:

9 $${{\mathscr{U}}}_{q,s}^{2}({\rho }_{A{B}_{1}{B}_{2}\cdots {B}_{N-1}})\ge {{\mathscr{U}}}_{q,s}^{2}({\rho }_{A{B}_{1}})+{{\mathscr{U}}}_{q,s}^{2}({\rho }_{A{B}_{2}})+\cdots +{{\mathscr{U}}}_{q,s}^{2}({\rho }_{A{B}_{N-1}}),$$

with $$(q,s)\in  {\mathcal R} $$.

*Proof*. The formula of ${{\mathscr{U}}}_{q,s}$ (5) cannot be applied to ${{\mathscr{U}}}_{q,s}({\rho }_{A{B}_{1}{B}_{2}\cdots {B}_{N-1}})$ since the subsystem *B*_1_*B*_2_ … *B*_*N*−1_ is not a logic qubit. However, We can apply the convex roof extension formula (2) of the pure state entanglement. Let ${\rho }_{A{B}_{1}{B}_{2}\cdots {B}_{N-1}}={\sum }_{k}\,{p}_{k}{|{\psi }_{k}\rangle }_{A{B}_{1}{B}_{2}\cdots {B}_{N-1}}\langle {\psi }_{k}|$ be the optimal decomposition that minimizes ${{\mathscr{U}}}_{q,s}({\rho }_{A{B}_{1}{B}_{2}\cdots {B}_{N-1}})$. Then we have

10 $$\begin{array}{rcl}{{\mathscr{U}}}_{q,s}^{2}({\rho }_{A{B}_{1}{B}_{2}\cdots {B}_{N-1}}) & = & {[\sum _{k}{p}_{k}{{\mathscr{U}}}_{q,s}({|{\psi }_{k}\rangle }_{A{B}_{1}{B}_{2}\cdots {B}_{N-1}})]}^{2}\\  & \mathop{=}\limits^{({\rm{a}})} & {[\sum _{k}{p}_{k}{f}_{q,s}({\mathscr{C}}({|{\psi }_{k}\rangle }_{A{B}_{1}{B}_{2}\cdots {B}_{N-1}}))]}^{2}\\  & \mathop{\ge }\limits^{({\rm{b}})} & {[{f}_{q,s}(\sum _{k}{p}_{k}{\mathscr{C}}({|{\psi }_{k}\rangle }_{A{B}_{1}{B}_{2}\cdots {B}_{N-1}}))]}^{2}\\  & \mathop{\ge }\limits^{({\rm{c}})} & {[{f}_{q,s}({\mathscr{C}}({\rho }_{A{B}_{1}{B}_{2}\cdots {B}_{N-1}}))]}^{2}={g}_{q,s}^{2}({{\mathscr{C}}}^{2}({\rho }_{A{B}_{1}{B}_{2}\cdots {B}_{N-1}})),\end{array}$$

where (a) follows from the pure state formula of the ${{\mathscr{U}}}_{q,s}$ and takes the ${f}_{q,s}({\mathscr{C}})$ as a function of concurrence ${\mathscr{C}}$ for $(q,s)\in  {\mathcal R}$; (b) is due to the fact that ${f}_{q,s}({\mathscr{C}})$ is a convex function of concurrence for $(q,s)\in  {\mathcal R}$; and (c) is due to the convexity of concurrence for mixed states.

$$\begin{array}{lll}{{\mathscr{U}}}_{q,s}^{2}({\rho }_{A{B}_{1}{B}_{2}\cdots {B}_{N-1}}) & \mathop{\ge }\limits^{({\rm{d}})} & {g}_{q,s}^{2}({{\mathscr{C}}}^{2}({\rho }_{A{B}_{1}{B}_{2}\cdots {B}_{N-1}}))\\  & \mathop{\ge }\limits^{({\rm{e}})} & {g}_{q,s}^{2}({{\mathscr{C}}}^{2}({\rho }_{A{B}_{1}})+{{\mathscr{C}}}^{2}({\rho }_{A{B}_{2}})+\cdots +{{\mathscr{C}}}^{2}({\rho }_{A{B}_{N}-1}))\\  & \mathop{\ge }\limits^{({\rm{f}})} & {g}_{q,s}^{2}({{\mathscr{C}}}^{2}({\rho }_{A{B}_{1}}))+{g}_{q,s}^{2}({{\mathscr{C}}}^{2}({\rho }_{A{B}_{2}}))+\cdots +{g}_{q,s}^{2}({{\mathscr{C}}}^{2}({\rho }_{A{B}_{N-1}}))\\  & = & {{\mathscr{U}}}_{q,s}^{2}({\rho }_{A{B}_{1}})+{{\mathscr{U}}}_{q,s}^{2}({\rho }_{A{B}_{2}})+\cdots +{{\mathscr{U}}}_{q,s}^{2}({\rho }_{A{B}_{N-1}})\end{array}$$

where (d) is from (10); (e) and (f) are due to Propositions 1 and 2, respectively.$\square$

**Remark 1**. *SU-*(*q*,*s*)-*E provides us the broad class of monogamy inequalities and recovers the monogamy relations for squared EOF, T*_*q*_-*E and R*_*q*_-*E for different values of q and s. Specifically*, (9) *can be reduced to the following monogamy relations*:

i. Squared EOF^6,10^, for *q* → 1
  11 $${{\mathscr{E}}}_{f}^{2}({\rho }_{A{B}_{1}{B}_{2}\cdots {B}_{N-1}})\ge {{\mathscr{E}}}_{f}^{2}({\rho }_{A{B}_{1}})+{{\mathscr{E}}}_{f}^{2}({\rho }_{A{B}_{2}})+\cdots +{{\mathscr{E}}}_{f}^{2}({\rho }_{A{B}_{N-1}}),$$
ii. Squared R_*q*_-E^19,20^, for *s* → 0
  12 $${ {\mathcal R} }_{q}^{2}({\rho }_{A{B}_{1}{B}_{2}\cdots {B}_{N-1}})\ge { {\mathcal R} }_{q}^{2}({\rho }_{A{B}_{1}})+{ {\mathcal R} }_{q}^{2}({\rho }_{A{B}_{2}})+\cdots +{ {\mathcal R} }_{q}^{2}({\rho }_{A{B}_{N-1}}),$$
iii. Squared T_*q*_-E^16–18^, for *s* → 1

13 $${{\mathscr{T}}}_{q}^{2}({\rho }_{A{B}_{1}{B}_{2}\cdots {B}_{N-1}})\ge {{\mathscr{T}}}_{q}^{2}({\rho }_{A{B}_{1}})+{{\mathscr{T}}}_{q}^{2}({\rho }_{A{B}_{2}})+\cdots +{{\mathscr{T}}}_{q}^{2}({\rho }_{A{B}_{N-1}})\mathrm{.}$$

### The *α*th power ${{\mathscr{U}}}_{q,s}$ monogamy relation

In this subsection, we establish the *α*th power ${{\mathscr{U}}}_{q,s}$ based general monogamy and polygamy inequalities.

**Theorem 2**. For an arbitrary tripartite qubit state ρ_A__1__A__2__A__3_, we have

14 $${{\mathscr{U}}}_{q,s}^{\alpha }({\rho }_{{A}_{1}{A}_{2}{A}_{3}})\ge {{\mathscr{U}}}_{q,s}^{\alpha }({\rho }_{{A}_{1}{A}_{2}})+{{\mathscr{U}}}_{q,s}^{\alpha }({\rho }_{{A}_{1}{A}_{3}}),$$

with *α* ≥ 2 and $$(q,s)\in  {\mathcal R} $$.

*Proof*. According to the monogamy relation given in (9)

$${{\mathscr{U}}}_{q,s}^{2}({\rho }_{{A}_{1}{A}_{2}{A}_{3}})\ge {{\mathscr{U}}}_{q,s}^{2}({\rho }_{{A}_{1}{A}_{2}})+{{\mathscr{U}}}_{q,s}^{2}({\rho }_{{A}_{1}{A}_{3}}),$$

for an arbitrary tripartite state *ρ*_*A*__1__*A*__2__*A*__3_ with $q\ge (\sqrt{9{s}^{2}-24s+28}-(2+3s))/(2(2-3s))$, 0 ≤ *s* ≤ 1 and $qs\le (5+\sqrt{13})/2$. If ${\rm{\min }}\{{{\mathscr{U}}}_{q,s}^{2}({\rho }_{{A}_{1}{A}_{2}}),{{\mathscr{U}}}_{q,s}^{2}({\rho }_{{A}_{1}{A}_{3}})\}=0$, the inequality (14) obviously holds. Without any loss of generality, we assume that ${{\mathscr{U}}}_{q,s}^{2}({\rho }_{{A}_{1}{A}_{2}})\ge {{\mathscr{U}}}_{q,s}^{2}({\rho }_{{A}_{1}{A}_{3}})$. Then, we have

$$\begin{array}{ccc}{{\mathscr{U}}}_{q,s}^{\alpha }({\rho }_{{A}_{1}{A}_{2}{A}_{3}}) & \ge  & {({{\mathscr{U}}}_{q,s}^{2}({\rho }_{{A}_{1}{A}_{2}})+{{\mathscr{U}}}_{q,s}^{2}({\rho }_{{A}_{1}{A}_{3}}))}^{\frac{\alpha }{2}}\\  & \mathop{\ge }\limits^{({\rm{a}})} & {{\mathscr{U}}}_{q,s}^{\alpha }({\rho }_{{A}_{1}{A}_{2}})(1+{(\frac{{{\mathscr{U}}}_{q,s}^{2}({\rho }_{{A}_{1}{A}_{3}})}{{{\mathscr{U}}}_{q,s}^{2}({\rho }_{{A}_{1}{A}_{2}})})}^{\frac{\alpha }{2}})\\  & = & {{\mathscr{U}}}_{q,s}^{\alpha }({\rho }_{{A}_{1}{A}_{2}})+{{\mathscr{U}}}_{q,s}^{\alpha }({\rho }_{{A}_{1}{A}_{3}}),\end{array}$$

where (a) comes from the algebraic inequality 1 + *β*^*γ*^ ≤ (1 + *β*)^*γ*^ for *β* ≤ 1, and *γ* ≥ 1.$\square$

**Theorem 3**. *The αth power* ${{\mathscr{U}}}_{q,s}$ satisfies the following polygamy relation for any tripartite state

15 $${{\mathscr{U}}}_{q,s}^{\alpha }({\rho }_{{A}_{1}{A}_{2}{A}_{3}}) < {{\mathscr{U}}}_{q,s}^{\alpha }({\rho }_{{A}_{1}{A}_{2}})+{{\mathscr{U}}}_{q,s}^{\alpha }({\rho }_{{A}_{1}{A}_{3}}),$$

with *α* ≤ 0 and $$(q,s)\in  {\mathcal R} $$.

*Proof*. For any tripartite state *ρ*_*A*__1__*A*__2__*A*__3_ with *α* ≤ 0, we have

$$\begin{array}{ccc}{{\mathscr{U}}}_{q,s}^{\alpha }({\rho }_{{A}_{1}{A}_{2}{A}_{3}}) & \le  & {({{\mathscr{U}}}_{q,s}^{2}({\rho }_{{A}_{1}{A}_{2}})+{{\mathscr{U}}}_{q,s}^{2}({\rho }_{{A}_{1}{A}_{3}}))}^{\frac{\alpha }{2}}\\  & \mathop{ < }\limits^{({\rm{a}})} & {{\mathscr{U}}}_{q,s}^{\alpha }({\rho }_{{A}_{1}{A}_{2}})(1+{(\frac{{{\mathscr{U}}}_{q,s}^{2}({\rho }_{{A}_{1}{A}_{3}})}{{{\mathscr{U}}}_{q,s}^{2}({\rho }_{{A}_{1}{A}_{2}})})}^{\frac{\alpha }{2}})\\  & = & {{\mathscr{U}}}_{q,s}^{\alpha }({\rho }_{{A}_{1}{A}_{2}})+{{\mathscr{U}}}_{q,s}^{\alpha }({\rho }_{{A}_{1}{A}_{3}}),\end{array}$$

where (a) follows from 1 + *β*^*γ*^ > (1 + *β*)^*γ*^ for *β* > 0, and *γ* ≤ 0.$\square$

**Remark 2**. *Theorem 2 and Theorem 3 have established the monogamy and dual monogamy inequalities for the αth power* ${{\mathscr{U}}}_{q,s}$ for *α* ≥ 2 and *α* ≤ 0, respectively in a tripartite scenario. These relations can be generalized for multiqubit systems by using induction and simple algebraic inequalities.

### Multipartite entanglement indicators based on the SU-(*q*, *s*)-E

From monogamy relation (9) of SU-(*q*,*s*)-E, we build a multipartite entanglement indicator that can be utilized to detect entanglement in the *N*-qubit state *ρ*_*A*__1_*A*_2__…_ *A*_*N*_. The indicator ${{\mathscr{J}}}_{q,s}$ is defined as

16 $${{\mathscr{J}}}_{q,s}({\rho }_{{A}_{1}{A}_{2}\cdots {A}_{N}})=\,min\sum _{i}\,{p}_{i}{{\mathscr{J}}}_{q,s}({|{\psi }_{i}\rangle }_{{A}_{1}|{A}_{2}\cdots {A}_{N}}),$$

where the minimization is performed over all pure state decompositions of *ρ*_*A*__1_*A*_2__…_ *A*_*N*_. This indicator essentially originates from the convex-roof of the pure state indicator ${{\mathscr{J}}}_{q,s}({|\psi \rangle }_{{A}_{1}|{A}_{2}\cdots {A}_{N}})={{\mathscr{U}}}_{q,s}^{2}({|\psi \rangle }_{{A}_{1}|{A}_{2}\cdots {A}_{N}})-{\sum }_{i\mathrm{=2}}^{N}\,{{\mathscr{U}}}_{q,s}^{2}({\rho }_{{A}_{1}{A}_{i}})$. Then it becomes

17 $${{\mathscr{J}}}_{q,s}({\rho }_{{A}_{1}{A}_{2}\cdots {A}_{N}})={{\mathscr{U}}}_{q,s}^{2}({\rho }_{{A}_{1}|{A}_{2}\cdots {A}_{N}})-\mathop{\sum }\limits_{i=2}^{N}\,{{\mathscr{U}}}_{q,s}^{2}({\rho }_{{A}_{1}{A}_{i}}),$$

which quantifies the residual entanglement in the system.

Following examples demonstrate the universal nature of ${{\mathscr{J}}}_{q,s}$ as an effective entanglement indicator. In particular, we evaluate (17) for the W-state, and for the state which is in the superposition of Greenberger-Horne-Zeilinger (GHZ) and W states. The nonzero values of ${{\mathscr{J}}}_{q,s}$ in these examples asserts its validity as a genuine entanglement indicator.

**Example 1**. *An N-qubit W-state is defined as*

18 $$|{W}_{N}\rangle =\frac{1}{\sqrt{N}}(|10\cdots 0\rangle +|01\cdots 0\rangle +\cdots +|0\cdots 01\rangle )\mathrm{.}$$

The indicator for the *N*-qubit W-class state can be written as

19 $${{\mathscr{J}}}_{q,s}(|{W}_{N}\rangle )={g}_{q,s}^{2}({{\mathscr{C}}}^{2}({\rho }_{{A}_{1}|{A}_{2}\cdots {A}_{N}}))-(N-1){g}_{q,s}^{2}({{\mathscr{C}}}^{2}({\rho }_{{A}_{1}{A}_{2}})),$$

where ${{\mathscr{C}}}^{2}({\rho }_{{A}_{1}|{A}_{2}\cdots {A}_{N}})=4(N-1)/{N}^{2}$ and ${{\mathscr{C}}}^{2}({\rho }_{{A}_{1}{A}_{2}})=4/{N}^{2}$. Via the established monogamy relation of the squared concurrence, the three tangle ${{\mathscr{J}}}_{{\mathscr{C}}}$ (genuine tripartite entanglement measure) is defined as^4^

20 $${{\mathscr{J}}}_{{\mathscr{C}}}^{2}({|\psi \rangle }_{ABC})={{\mathscr{C}}}^{2}({\rho }_{A|BC})-{{\mathscr{C}}}^{2}({\rho }_{AB})-{{\mathscr{C}}}^{2}({\rho }_{AC})\mathrm{.}$$

**Figure 1:**
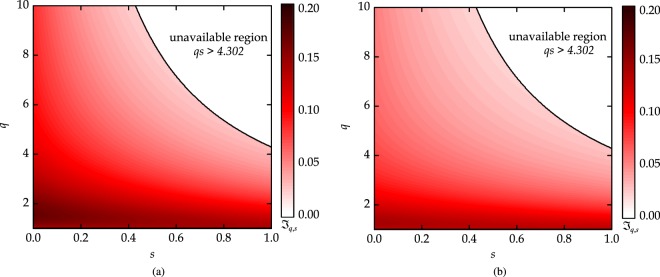
The indicator ${{\mathscr{J}}}_{q,s}$ results for W-state with (**a**) *N* = 4, and (**b**) *N* = 5. The solid black line shows the boundary *qs* = 4.302. Non zero values show the residual entanglement in the system.

The three tangle cannot detect the tripartite entangled W-state^4^. However, the indicator ${{\mathscr{J}}}_{q,s}$ efficiently detects the entanglement in this state. We plot the indicator as a function of (*q*,*s*) for the four and five qubit W-state in Fig. 1. The indicator has nonzero values when entanglement is present in the system.

**Example 2**. *We consider a superposition state of GHZ state and the W-state*

21 $${|\psi \rangle }_{ABC}=\sqrt{p}|GHZ\rangle -\sqrt{1-p}|W\rangle ,$$

where $$|GHZ\rangle =\frac{1}{\sqrt{2}}({|0\rangle }^{\otimes 3}+{|1\rangle }^{\otimes 3})$$ and $$|W\rangle =\frac{1}{\sqrt{3}}(|001\rangle +|010\rangle +|100\rangle ).$$

**Figure 2:**
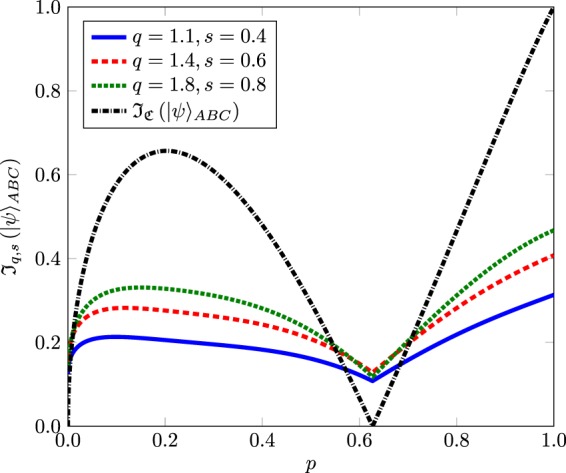
The indicator ${{\mathscr{J}}}_{q,s}$ for superposition of GHZ and W-state with *q* = 1.8, *s* = 0.8 (dotted green line), *q* = 1.4, *s* = 0.6 (dashed red line), and *q* = 1.1, *s* = 0.4 (solid blue line). The three tangle ${{\mathscr{J}}}_{{\mathscr{C}}}$ of ${|\psi \rangle }_{ABC}$ is also shown with dashdotted black line. ${{\mathscr{J}}}_{q,s}$ is positive for these value of *q* and *s*, but ${{\mathscr{J}}}_{q,s}^{2}$ is zero for *p* = 0, and *p* = 0.627.

The three tangle of ${|\psi \rangle }_{ABC}$ is ${{\mathscr{J}}}_{{\mathscr{C}}}({|\psi \rangle }_{ABC})=\mathrm{(9}{p}^{2}-8\sqrt{6}\sqrt{p{\mathrm{(1}-p)}^{3}})/9$ and is zero for *p* = 0, and *p* = 0.627^6,24^. This shows some flaw in the entanglement indicator. In this scenario, ${{\mathscr{J}}}_{q,s}$ multipartite entanglement indicator shown in (17) is used. The value of ${{\mathscr{J}}}_{q,s}({|\psi \rangle }_{ABC})$ is calculated through the analytic formula of the ${{\mathscr{U}}}_{q,s}$ for bipartite states. There is no need for convex-roof for the pure state. In Fig. 2, we draw the comparison between the ${{\mathscr{J}}}_{{\mathscr{C}}}$ and ${{\mathscr{J}}}_{q,s}$. We can see that ${{\mathscr{J}}}_{q,s}$ is positive for all values of *p*.

## Discussion

Unified-(*q*,*s*) entanglement is a two-parameter class of well defined bipartite entanglement measures. The generalized analytic formula of ${{\mathscr{U}}}_{q,s}$ has been proved for the region $(q,s)\in  {\mathcal R}$, which encompasses EOF^5^, Tsallis-*q* entanglement^16–18^ and Renyi-*q* entanglement^19,25^ as its special cases. We have investigated the monogamy relation for SU-(*q*,*s*)-E, which classifies the entanglement distribution in multipartite systems. The monogamy relation of SU-(*q*,*s*)-E enables us to construct an indicator, which overcomes all known flaws and detects genuine multipartite entanglement better than previously known indicators. This superior performance in the detection of multiqubit states is exemplified on W-class states and compared with concurrence based entanglement indicator. The established monogamy relation gives the nontrivial and computable lower bound for the ${{\mathscr{U}}}_{q,s}$. Furthermore, we also proved the *α*th power ${{\mathscr{U}}}_{q,s}$ based general monogamy and polygamy relations. In summary, the results in this paper provide the unified monogamy relations of multipartite entanglement, covering several previous results as its special cases.

## Methods

### ${f}_{q,s}({\mathscr{C}})$ is a convex function of the concurrence ${\mathscr{C}}$

We prove the convexity of *f*_*q*,*s*_(*x*) in the extended region $q\ge (\sqrt{9{s}^{2}-24s+28}-(2+3s))$/(2(2 − 3*s*)), 0 ≤ *s* ≤ 1, and $qs\le (5+\sqrt{13})/2$, which was previously shown for the region 1 ≥ *s* ≥ 0 and 3/*s* ≥ *q* ≥ 1. We consider the second-order derivative of *f*_*q*,*s*_(*x*) for 1 > *q* > 0 and *qs* ∈ (3, 5), respectively.

For the region 0 < *q* < 1, we graphically analyze the solution of ${\partial }^{2}{{\mathscr{U}}}_{q,s}({\mathscr{C}})/\partial {x}^{2}=0$. It can be shown that for fixed *s* ∈ [0,1], the value of x to keep the second derivative nonnegative increases monotonically with *q*^18,25^. Therefore, the critical point exists under the limit *x* → 1. We apply limit *x* → 1 to obtain the critical point of *q*. After applying the limit and some simplification, we have

22 $$-\frac{{2}^{s-qs}q(3+{q}^{2}(3s-2)-q(3s+2))}{3}=0,$$

which gives the critical point is ${q}_{\ast }=(\sqrt{9{s}^{2}-24s+28}-(2+3s))/(2(2-3s))$ with 0 ≤ *s* ≤ 1 for the region 0 < *q* < 1. The second-order derivative is always nonnegative when $q > {q}_{\ast }$.

For *qs* ∈ (3, 5), we select *qs* ≤ 4.302 because when *s* → 1, *f*_*q*,*s*_(*x*) approaches to the Tsallis entropy for which the second derivative is known to be nonnegative for *q* ≤ 4.302^18^. For the analytical proof, we define a new range of *s* on the basis of this constraint, that is, 0 ≤ *s* ≤ *min*{4.302/*q*,1}. We enforce this constraint by substituting *s* = 4.302/*q* in the expression for the second derivative of *f*_*q*,*s*_(*x*). In the following, we prove that the second derivative is nonnegative for *q* ≥ 4.302. The second derivative of *f*_*q*,*s*_(*x*) after its simplification is

23 $$\begin{array}{ccc}\frac{{{\rm{\partial }}}^{2}{f}_{q,s}(x)}{{\rm{\partial }}{x}^{2}} & \ge  & (q-4.302){x}^{2}\sqrt{1-{x}^{2}}{({B}^{q-1}-{A}^{q-1})}^{2}\\  &  & +({A}^{q}+{B}^{q})[({B}^{q-1}-{A}^{q-1})-{x}^{2}\sqrt{1-{x}^{2}}(q-1)({A}^{q-2}+{B}^{q-2})],\end{array}$$

where $A=(1-\sqrt{1-{x}^{2}})$ and $B=(1+\sqrt{1-{x}^{2}})$. First, we apply the binomial expansion on *A*^*q*−1^ and *B*^*q*−1^ to write

24 $$({B}^{q-1}-{A}^{q-1})\ge 2(q-1)\sqrt{1-{x}^{2}}.$$

Substituting (24) into (23), we get

25 $$\begin{array}{ccc}\frac{{{\rm{\partial }}}^{2}{f}_{q,s}(x)}{{\rm{\partial }}{x}^{2}} & \ge  & (q-4.302){x}^{2}\sqrt{1-{x}^{2}}{(2(q-1)\sqrt{1-{x}^{2}})}^{2}\\  &  & +\sqrt{1-{x}^{2}}({A}^{q}+{B}^{q})(q-1)[2-{x}^{2}({A}^{q-2}+{B}^{q-2})].\end{array}$$

Using the inequality of arithmetic and geometric, i.e., $x+y\ge 2\sqrt{xy}$, we obtain

26 $$\begin{array}{rcl}{A}^{q-2}+{B}^{q-2}\ge 2\sqrt{{(AB)}^{q-2}} & = & 2{Z}^{q-2},\\ {A}^{q}+{B}^{q}\ge 2\sqrt{{(AB)}^{q}} & = & 2{Z}^{q},\end{array}$$

where *AB* = *Z*^2^. Substituting (26) in (23) and after some manipulations, we finally obtain the inequality:

27 $$\frac{{{\rm{\partial }}}^{2}{f}_{q,s}(x)}{{\rm{\partial }}{x}^{2}}\ge 4(q-1)\sqrt{1-{x}^{2}}[{x}^{2}\sqrt{1-{x}^{2}}(q-4.302)(q-1)+{x}^{q}(1-{x}^{q})].$$

Now we can see that if *q* ≥ 4.302 then (27) is positive and the upper constraint *qs* ≤ 4.302 is satisfied. The second derivative is nonnegative for *qs* ≤ 4.302 when 0 ≤ *s* ≤ 1.

### ${g}_{q,s}^{2}({{\mathscr{C}}}^{2})$ is an increasing monotonic function of the squared concurrence ${{\mathscr{C}}}^{2}$

Note that we can rewrite the Eq. (5) as

28 $${{\mathscr{U}}}_{q,s}({|\psi \rangle }_{AB})={g}_{q,s}({{\mathscr{C}}}^{2}({|\psi \rangle }_{AB})),$$

where

29 $${g}_{q,s}(x)=\frac{{({\beta }_{+}^{q}+{\beta }_{-}^{q})}^{s}-{2}^{qs}}{(1-q)s{2}^{qs}}$$

where ${\beta }_{\pm }=(1\pm \sqrt{1-x})$. We investigate the monotonicity of ${g}_{q,s}^{2}(x)$, since the SU-(*q*,*s*)-E is a monotonically increasing function of ${{\mathscr{C}}}^{2}$ if *dg*_*q*,*s*_^2^(*x*)/*dx* > 0 with $x={{\mathscr{C}}}^{2}$. After some calculation, we have

30 $$\frac{{\rm{\partial }}{g}_{q,s}^{2}(x)}{{\rm{\partial }}x}=M{2}^{-2qs}(-{2}^{qs}+{({F}^{q}+{E}^{q})}^{s})[\frac{q{s}^{2}{({F}^{q}+{(E)}^{q})}^{s-1}({F}^{q-1}-{E}^{q-1})}{\sqrt{1-x}}],$$

where *M* = 1/(*q*−1)^2^, $E=\mathrm{(1}+\sqrt{1-x})$, $F=\mathrm{(1}-\sqrt{1-x})$. The derivative (30) is non-negative for *q* ≥ 0 and 0 ≤ *x* ≤ 1. Thus ${g}_{q,s}^{2}({{\mathscr{C}}}^{2})$ is a monotonically increasing function.

### ${g}_{q,s}^{2}({{\mathscr{C}}}^{2})$ is a convex function of the squared concurrence ${{\mathscr{C}}}^{2}$

The SU-(*q*,*s*)-E is convex in ${{\mathscr{C}}}^{2}$ when the second order derivative ${{\rm{\partial }}}^{2}{{\mathscr{U}}}_{q,s}^{2}(x)/{\rm{\partial }}{x}^{2}$  ≥ 0 where $x={{\mathscr{C}}}^{2}$. We define function,

31 $${Z}_{q,s}(x)=\frac{{{\rm{\partial }}}^{2}{g}_{q,s}^{2}(x)}{{\rm{\partial }}{x}^{2}}$$

on the domain $D=\{(x,s,q)\mathrm{|0}\le x\le \mathrm{1,0}\le s\le \mathrm{1,}(\sqrt{9{s}^{2}-24s+28}-(2+3s))/(2(2-3s))\le q\le \mathrm{4.302/}s\}$.

After some calculation, we have

32 $${Z}_{q,s}(x)=q{s}^{2}{2}^{-2qs}M{A}^{s-2}\,[\frac{\{A({2}^{qs}-{A}^{s})(B-(q-1)\sqrt{1-x}({F}^{-2+q}+{E}^{-2+q}))\}+{B}^{2}\sqrt{1-x}\{q(1-s){2}^{qs}+q(2s-1){A}^{s}\}}{2{(1-x)}^{3/2}}]$$

where *A* = (*F*^*q*^ + *E*^*q*^) and *B* = (*E*^*q*−1^ − *F*^*q*−1^).

The intermediate value theorem states that if a continuous function has values of opposite sign inside a domain, then it has a root in that domain. The function *Z*_*q*,*s*_(*x*) is continuous on the domain *D*. We divide *D* into two sub domains,

$${D}_{1}=\{(x,s,q)\mathrm{|0}\le x\le \mathrm{1,}\,0\le s\le \mathrm{1,}(\sqrt{9{s}^{2}-24s+28}-(2+3s))/(2(2-3s))\le q\le 1\},$$

and

$${D}_{2}=\{(x,s,q)|0\le x\le 1,0\le s\le 1,1\le q\le 4.302/s\}.$$

**Figure 3:**
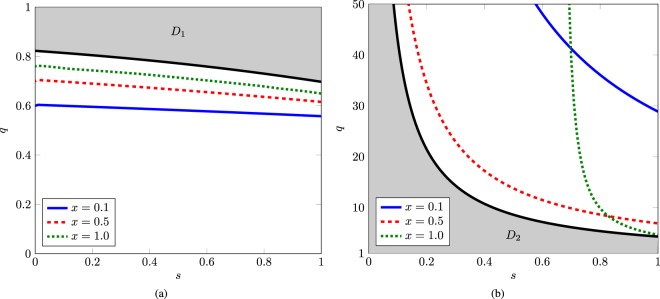
Domain (**a**) *D*_1_, and (**b**) *D*_2_ are shown as shaded region. Solid black lines show the domain boundary and blue, green, and red lines indicate the roots of *Z*_*q*,*s*_(*x*) for different values of *x*.

**Figure 4:**
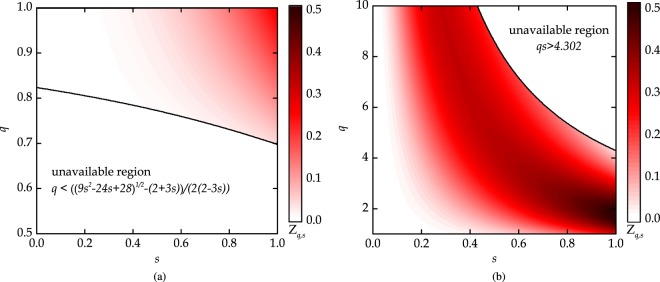
The positivity of *Z*_*q*,*s*_(*x*) for *x* → 1 on the domain (**a**) *D*_1_, and (**b**) *D*_2_.

We plot the solution of *Z*_*q*,*s*_(*x*) = 0 for different values of *x*. As shown in Fig. 3, no root of *Z*_*q*,*s*_(*x*) exists inside the domain *D*. Thus, all values of *Z*_*q*,*s*_(*x*) on the domain *D* have the same sign. This means that if *Z*_*q*,*s*_ is positive for any value of *x* in *D*, then it is positive on the entire domain *D*. We have plotted the function *Z*_*q*,*s*_(*x*) on the domain *D* in Fig. 4 for *x* → 1. The function *Z*_*q*,*s*_(*x*) is positive on the domain *D*. This means that the second derivative is positive, therefore ${g}_{q,s}^{2}(x)$ is convex on the domain *D*. Therefore, ${g}_{q,s}^{2}({{\mathscr{C}}}^{2})$ is convex function of the squared concurrence ${{\mathscr{C}}}^{2}$.
